# Configurational diffusion transport of water and oil in dual continuum shales

**DOI:** 10.1038/s41598-021-81004-1

**Published:** 2021-01-25

**Authors:** Mohammed Abdul Qadeer Siddiqui, Filomena Salvemini, Hamed Lamei Ramandi, Paul Fitzgerald, Hamid Roshan

**Affiliations:** 1 School of Minerals and Energy Resources Engineering, UNSW Australia, Kensington, Sydney, 2052 Australia; 2 Australian Nuclear Science and Technology Organisation (ANSTO), Lucas Heights, NSW 2234 Australia; 3 Sydney Analytical, Core Research, The University of Sydney, Sydney, NSW 2006 Australia

**Keywords:** Crude oil, Natural gas

## Abstract

Understanding fluid flow in shale rocks is critical for the recovery of unconventional energy resources. Despite the extensive research conducted on water and oil flow in shales, significant uncertainties and discrepancies remain in reported experimental data. The most noted being that while oil spreads more than water on shale surfaces in an inviscid medium, its uptake by shale pores is much less than water during capillary flow. This leads to misjudgement of wettability and the underlying physical phenomena. In this study, therefore, we performed a combined experimental and digital rock investigation on an organic-rich shale including contact angle and spontaneous imbibition, X-ray and neutron computed tomography, and small angle X-ray scattering tests to study the potential physical processes. We also used non-equilibrium thermodynamics to theoretically derive constitutive equations to support our experimental observations. The results of this study indicate that the pre-existing fractures (first continuum) imbibe more oil than water consistent with contact angle measurements. The overall imbibition is, however, higher for water than oil due to greater water diffusion into the shale matrix (second continuum). It is shown that more water uptake into shale is controlled by pore size and accessibility in addition to capillary or osmotic forces i.e. configurational diffusion of water versus oil molecules. While the inorganic pores seem more oil-wet in an inviscid medium, they easily allow passage of water molecules compared to oil due to the incredibly small size of water molecules that can pass through such micro-pores. Contrarily, these strongly oil-wet pores possessing strong capillarity are restricted to imbibe oil simply due to its large molecular size and physical inaccessibility to the micro-pores. These results provide new insights into the previously unexplained discrepancy regarding water and oil uptake capacity of shales.

## Introduction

### Water and oil uptake by shales

Shales are tight rocks with complex properties^1,2^. Understanding water and oil flow in shales is critical due to its direct consequence in high fluid loss during hydraulic fracturing which is a major environmental and technical concern^3,4^. There has been a widespread discrepancy observed in many shales around the world. In presence of air (inviscid medium), oil spreads more than water on a shale’s surface, whereas its capillary uptake by the shale pores is significantly lower than that of water. This has led to incomplete understanding of water and oil diffusion mechanisms in shales igniting research interest in the fundamental-level causes of such an intriguing phenomenon. Implicating water reactivity with clays and consequent micro-fracture initiation as the sole reason for higher water uptake was confuted in a recent study^5^. Therein, it was revealed that even when micro-fracture initiation was avoided with confinement, water uptake by shale was still much higher than oil uptake.

The water and oil spreading is usually assessed by contact angle (CA) measurements and the uptake capacities by spontaneous imbibition (SI) tests^6–8^ i.e. when capillarity is considered as the sole driving force of imbibition, the process is referred to as spontaneous imbibition^9^. It is usually not known whether the water or oil droplets during CA measurements on shale are in a Wenzel state, Cassie–Baxter state, or a meta-stable Cassie–Baxter state^10^. What is clear is that the complete spreading of oil (close to zero CA) does imply its attainment of the lowest energy state with no energy barriers remaining to be overcome^11^. On the same sample in an inviscid medium, slight water repellence (acute CA) implies that there are active energy barriers associated with the water droplet. This is an important implication since during capillary flow, the porous surface area is not polished, and hence offers energy barriers for the water and oil to overcome. It is, however, interesting to note that the effect of such energy barriers on the change in CAs (and hence its influence during capillary flow) may not be significant^11,12^. Therefore, the stark difference in water and oil uptake capacities of shale pores requires an explanation beyond the concept of wetting states and energy barriers. We postulate and later confirm the existence of configurational diffusion in the shale pore system as another controlling mechanism of flow.

### Configurational diffusion

Imbibition is a ubiquitous phenomenon pertaining to physics of fluids in porous media e.g. during multi-phase flow in rocks. While SI is generally implied to mean the uptake of fluids, in the jargon of molecular dynamics, the physics of this uptake process (as an exclusive effect of capillarity) is classified as diffusion in a mathematical sense. Depending on the type of porous media or that of diffusing fluids, diffusion can be unique or a combination of ordinary diffusion, Knudsen diffusion, surface diffusion, shape-changeable diffusion, and configurational diffusion^13–15^. More specifically, the ratio of pore size to the size of diffusing molecule dictates the diffusion mechanism^16^.

Amongst the various diffusion regimes, configurational diffusion is the least understood with no well-defined theory or diffusivity equations. The other regimes, on the other hand, are well described in literature with accurate diffusivity estimation theories available. Generally, only a fluid molecule with a size smaller than the pore opening can diffuse through that pore. Configurational diffusion, also referred to as hindered or restricted diffusion, is when molecular sizes of diffusing fluids are appreciably larger than the pore opening resulting in denial of entry into such pores^16–18^. Configurational diffusion has been widely observed in varying kinds of porous media^17,19–21^. For instance, the diffusivity of methane (CH_4_) was found to decrease drastically by several orders of magnitude below a limiting pore size of ~ 4 Å^16,22^. When the pore size and size of diffusing molecule are comparable, the molecule cannot escape the force field at the pore wall leading to dominant steric effects. Such steric effects cause sudden and drastic reduction in diffusivity of the molecule^16^. The existence of configurational diffusion was realized and extensively studied by chemical scientists for analysing the flow of fluids through zeolite catalysts^16,17^. Pioneering and ensuing subsurface porous media scientists, however, continued developing analytical models to predict SI in porous media seemingly without considering the concept of configurational diffusion as an important controlling mechanism^23–27^. Consequently, unexplained experimental deviations from their developed analytical models still exist especially for complex fluids^28^ and tight and heterogenous porous media such as shales^29^. Experimental investigation of configurational diffusion in geomaterials, however, offers significant challenges. Amongst the available experimental techniques, digital methods seem to be able to provide valuable information for such investigations.

### Digital methods

It has already been known that apart from capillarity, diffusion (chemical in nature) is the additional dominant mechanism responsible for higher water uptake in shales^5,30^. Based on the complete spreading of oil on shale surface, capillarity action must be stronger for oil than water. Gravimetric fluid uptake studies, however, fail to distinguish between the dominant uptake mechanisms for water and oil respectively for many cases. The slopes of weight increment curves have been often used to interpret overall uptake of fluids. Nevertheless, due to the tight coupling between different mechanisms in play, it cannot be ascertained how accurate these interpretations are. Such uncertainties pertaining to fluid flow in rocks are inherently due to the inability to visualize what occurs in-situ at a point-in-time and space^31^. In this regard, imaging techniques have gained profound prominence^32–34^. However, such imaging is marred with limitations despite the progress made in the enhancement of image quality and improvement of imaging resolutions^35–39^. For instance, to distinguish between phases, tracers are usually required whereas such tracers can themselves induce unwanted effects^40^. Such limitations have been overcome using neutron imaging because of the strong attenuation of neutrons by hydrogen atoms^39,40^. Hydrogen-rich fluids such as water and oil can hence be easily tracked and distinguished from other phases without using tracers.

In neutron imaging, neutrons are transmitted when they pass through an object. The intensity of transmitted neutrons forms the basis for neutron imaging^40^, in which both 2D and 3D images can be produced. For instance, DiStefano et al.^41^ studied the height of spontaneous water rise in Eagle Ford shale fractures using 2D neutron radiographs. Similarly, Roshankhah et al.^42^ used both 2D radiographs and 3D tomograms to investigate the generation and propagation of hydraulic fractures in Marcellus shale. Recently, the potential of dual X-ray and neutron tomography techniques was demonstrated on a Middle Eastern shale to distinguish between different phases such as fractures, kerogens, and other minerals^43^. Similarly, neutron imaging techniques were also used for conventional rocks like sandstones and limestones^39,44^. In a more recent study, the dual potential of X-ray and nuclear techniques was highlighted using a novel data-driven approach to acquire quality resolution images using magnetic resonance imaging (MRI)^39^. There have been other novel attempts to characterize molecular diffusion paths using nuclear resonance techniques such as pulsed field gradient (PFG) nuclear magnetic resonance (NMR) in combination with magic-angle spinning (MAS)^45,46^. Such newer (NMR imaging) techniques have potential to overcome the limitations of independent X-ray or neutron imaging. It is noted that, to date, spatial resolution lower than ~ 3 µm could not be achieved on tested core specimens^47^. However, at a specific resolution, neutron imaging can detect the coalescence of hydrogen-rich water or oil molecules larger than the resolution. In the resulting tomography and typical greyscale colour coding (higher the neutron attenuation, brighter the grey tone), pores or void spaces appear as dark regions and hydrogen-rich molecules as bright ones. In addition to the instrument resolution, the detection of hydrogen molecules by neutron imaging is also related to the contrast resolution. Among other factors, the contrast resolution is dependent on the difference in attenuation between phases of interest in the samples. The phase must give a contrast beyond the intensity noise caused by counting statistic and background intensity in order to be detected. The as grey tone of each pixel is the visual representation of the weighted average of the contributions of the attenuation coefficients of the phases present in that pixel (or voxel equivalent in 3D). As a general empiric rule, the amount of hydrogen molecules to be present in a pixel should be such to cause a variation in attenuation coefficient between the matrix and the pore-filled water of at least 15% in order to be detected. If the composition of the matrix is well characterised, that can be theoretically determinate. However, there are limitations in the quantification capabilities of the standard neutron imaging methods due to neutron scattering and other systematic biases in scintillator-camera based detectors etc., thus causing deviations from the conventional description and measurement of the linear attenuation through the Beer Lambert law. Therefore, a reliable quantitative estimate of the hydrogen content is not achievable based only on the neutron attenuation coefficient (or grey tone) recoded for a pixel/voxel via tomographic measurement. It is thus efficient to utilize neutron imaging along with X-ray imaging. The fractures/macro-pores can be easily identified from X-ray and when superimposed on neutron images, water or oil in those fractures i.e. their molecular coalescence in the matrix can be quantified accurately. It is noted that X-ray tomography does not intend to measure the shale pore system but rather imaging the fractures/microfractures. On the other hand, neutron tomography can additionally track the hydrogen atoms even below its nominal voxel resolution.

This study is thus based on utilizing the dual potential of X-ray and neutron tomography techniques to digitally investigate configurational diffusion in shales that could explain the difference in water and oil uptakes i.e. X-ray imaging was used to map the internal micro-fractures and neutron imaging was employed to track the hydrogen atoms both in the fractures and the matrix and that the imaging of shale pores are not the intension of the imaging study. Hence, two organic-rich shale samples have been subjected to unconfined water and oil uptake with initial 3D X-ray and timely neutron imaging been used to provide insights into their respective uptake dynamics. Pore size characterization techniques including Small Angle X-ray Scattering (SAXS) and Focused Ion Beam–Scanning Electron Microscopy (FIB–SEM) have been used to support the conclusions. Also, theoretical corroboration of the configurational diffusion of water and oil in shales has been developed to support the experimental observations.

## Experimental materials and methods

### Shale and fluid samples

Organic-rich shale (ORS) samples from the Middle Velkerri Formation (MVF), Beetaloo sub-basin located in the state of Northern Territory, Australia, were used in this study. Details of the ORS’s petrophysical properties were published previously^48^. LECO CN analysis showed that it contained 3.15 wt% total organic carbon (TOC) content. X-ray diffraction (XRD) analysis found major clay minerals present to be illite-muscovite (15.7 wt%) and illite–smectite (7.4 wt%). Through mercury intrusion, it was found that matrix porosity was as low as 0.84%. Also, significant layering and micro-fractures along weak planes were found to exist in the MVF samples^48^.

The fluids used in this study for contact angle and fluid uptake tests were de-ionized water (DIW) with a resistivity of 18.2 MΩ cm at 25 °C and an isoparaffinic solvent (Soltrol-130) procured from Chevron Phillips Chemical Company. Soltrol-130 is made up of C_12_–C_14_ iso-alkane and was used as the oil-phase. It had a dynamic viscosity of 1.14 cP and specific gravity of 0.76. Henceforth, DIW and Soltrol-130 are simply referred to as water and oil respectively. DI water was used to serve as a base case because it has the maximum influence on the extent of clay induced uptake. The extent of clay-fluid interactions is readily altered with the addition of different salts making the judgment very subjective if used. Different salts present in water behave differently with different clay minerals at different concentrations e.g. KCl has non-reactivity with clay minerals. This would not allow for exact diagnosis of pore-size related configurational diffusion.

### Contact angle (CA) and spontaneous imbibition (SI)

The CA measuring setup consisted of a high definition Basler acA1600 20gm GigE camera with a Sony ICX274 charge-coupled device (CCD) sensor. Such a combination provides two-megapixel resolution and delivers 20 frames per second. Digital images were captured using the GigE connection to a computer and a designated Pylon software. CAs were measured using the ImageJ software (DropSnake plugin)^49^. A constant drop volume of ~ 6 μL was used for consistency and to ensure repeatability three drops were made at different locations of the shale surface. Before making the droplet, the surface was polished stepwise up to 1200-grit sandpaper and then rinsed with isopropanol. The polishing time was kept constant for consistency. Average CA was reported for the three drops including the average of left and right CAs.

**Figure 1:**
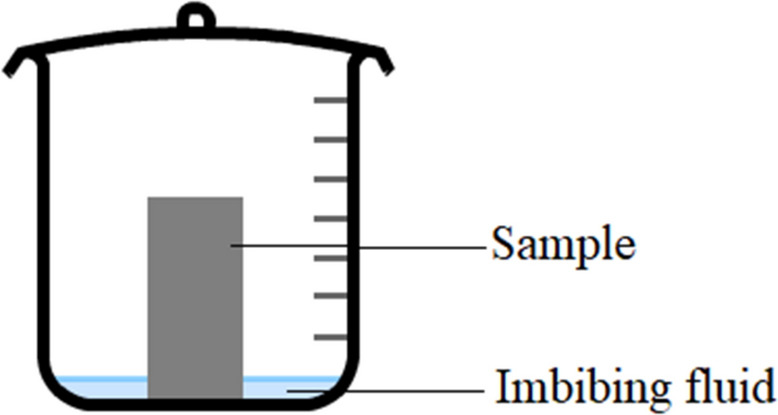
Schematic of water/oil imbibition setup.

For SI, two small cores (12.5 mm diameter × 26 mm length) were drilled parallel to bedding planes from a larger ORS sample (henceforth referred to as ORS1 and ORS2). The cores were then oven-dried at 105 °C for 24 h, then cooled in a desiccator to room temperature, and dry weighted. ORS1 was then exposed to water and ORS2 to oil from one of their faces (Fig. 1). The cores were removed from the water/oil bath, their surfaces wiped with a damp towel, and weighted using a weighting balance (Sartorius Entris323-1S; 0.0001 g accuracy). This process was repeated until no more apparent change was observed in sample weights. To quantitatively characterize imbibition, percent increments in sample weights (g) with respective to their original weights (g) was used as shown in Eq. (1)^5^.

1 $$\% increase\; in\; weight = \frac{{W_{t} \left( {\text{g}} \right) - W_{original} \left( {\text{g}} \right)}}{{W_{original} \left( {\text{g}} \right)}} \times 100$$

where $W_{t}$ is the weight of the sample after a certain imbibition time $t$ and $W_{original}$ is the original sample’s weight.

### X-ray computed tomography

**Figure 2:**
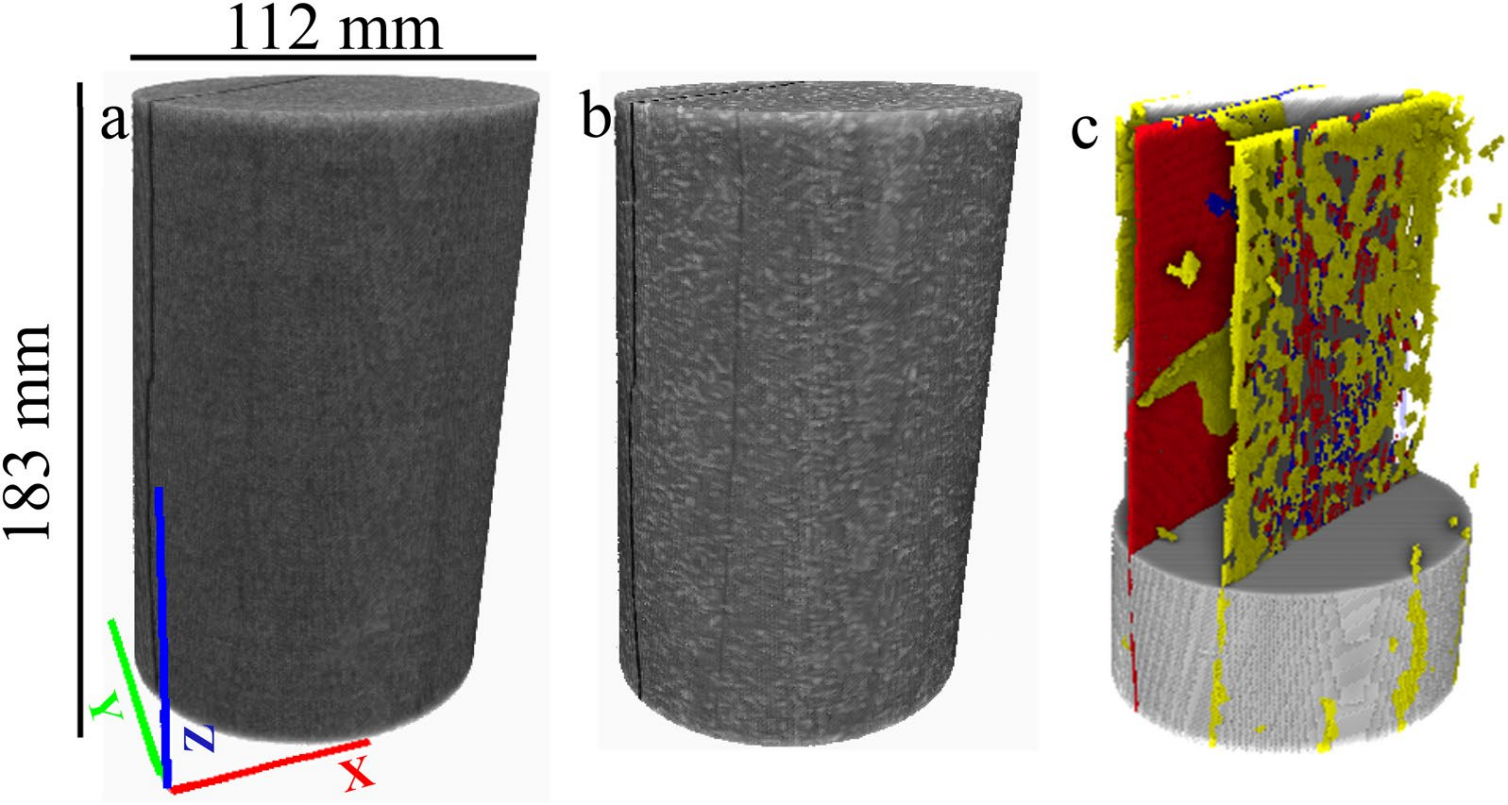
3D representations of ORS: (**a**) the greyscale neutron tomography image (black = fractures/micro-fractures, grey = solid matrix, (**b**) the grey-scale µ-CT image (black = fractures/micro-fractures, grey and white = organic and inorganic minerals), and (**c**) segmented image (grey = solid phase, red: empty fractures, blue = fluid-saturated fracture spaces and yellow = liquid diffused into the matrix adjacent to micro-fractures).

The samples were imaged using a high-resolution X-ray micro-computed tomography (µ-CT) scanner in dry condition. The scanner uses helical scanning technology to produce images with high fidelity and signal to noise ratio. The scanner obtains a series of projections (radiographs) from the sample along a helical trajectory at different viewing angles. The projections specify the cumulative attenuation of the X-ray beam through the sample, more details about the scanner and its specifications can be found elsewhere^50^. A reconstruction algorithm based on Katsevich^51^ is then used to reconstruct a 3D image (tomogram) from the projections. The reconstructed 3D tomogram is generally displayed in a 16-bit greyscale image. Each data point in the 3D image represents the effective X-ray attenuation coefficient of the sample at that point. The µ-CT image resolution/voxel size was 6.2 µm for both samples (Fig. 2a). To enhance the greyscale µ-CT image quality, anisotropic diffusion (AD) and unsharp mask (UM)^52^ filters were used. AD filter merges regions of similar grey-scale values and intra-region smoothing is promoted over edge smoothing. AD filter often causes some degree of blurring, which is reduced by UM filter. It has been shown that the application of AD and UM is highly effective for fractured rocks^53^. It is noted that the µ-CT imaging was conducted to map the internal structure of the micro-fractures in shale samples. This combined with neutron imaging will later assist us to investigate the water/oil distribution in micro-fractures.

### Neutron tomography

Neutrons have unique properties compared to other sub-atomic particles, i.e. electrons or protons. They are uncharged, can penetrate deeply into matter, and interact with the nucleus of an atom rather than with the diffuse electron cloud. Neutrons are also attenuated significantly by hydrogen compared with most other elements^54^. Therefore, the hydrogen-rich matter, e.g. water or oil in the fractures and pores of ORS1 and ORS2 can be easily distinguished from abiotic non-hydrogenous porous media and its contents^40,43^. ORS1 and ORS2 were neutron scanned in 3D at dry conditions initially after X-ray scanning, and then thrice during SI tests (after taking out from water/oil baths respectively) after 1 day, 4 days, and 13 days.

In this study, the neutron imaging was conducted on the DINGO beamline^55^ at OPAL research reactor in the Australian Nuclear Science and Technology Organization (ANSTO), Sydney. The instrument was set with the Photometrics 5 Megapixel (5056 × 2968) IRIS CMOS camera. In order to yield image at a pixel size of 13.6 µm, a 100 mm lens was coupled with a 0.02 mm thick Gd_2_O_2_S:Tb scintillation screen, thus resulting in a field of view of about 70 × 41 mm^2^. The tomographic scan consisted of 1800 angular projections, covering a range of 180°, and of 3 accumulations with an exposure time of 15 s acquired at each step angle. This was done to improve the image quality since the sum of short time radiographs produces higher signal-to-noise ratio (SNR) when added together than the equivalent longer exposure. The total acquisition time for one sample was around 24 h. The projections were first treated by applying an outlier removal filter with a 5-pixel radius and threshold of 50. Then the accumulations were summed up for each step angle. Flat field normalization with dose correction and dark current subtraction were applied. The data were processed using the Octopus code for tomographic reconstruction^56^, and the obtained slices were recomposed and evaluated using the Avizo 9.7.0 software (Fig. 2b). The tomographic reconstructions were further de-noised by applying AD filter.

### Image registration and segmentation techniques

To perform a voxel-by-voxel comparison of the images obtained using neutron tomography and µ-CT techniques and to highlight the 3D distribution of water or oil in the shale samples, all the images obtained from each sample were registered. For registration, a 3D image registration technique developed by Latham et al.^35^ was employed. The technique solves a transformation problem with 7 degrees of freedom using a multi-resolution optimisation search strategy and provides a pair of images with identical dimensions. Since the neutron tomography images were obtained at a resolution of 13.6 µm, the voxel size of micro-CT images was altered from 6.2 µm to 13.6 µm to obtain images with an identical voxel size allowing for a voxel-by-voxel comparison specifically in the fractures. The subsequent digital rock analyses were thus performed at a resolution of 13.6 µm; however, where high-resolution information was required high-resolution µ-CT images were used.

The registered images were then used for image segmentation, i.e. the process of classifying the greyscale images into two or more phases that are homogeneous with respect to some characteristic^50^. Converging active contours (CAC) was used for image segmentation of images. The method uses the gradient and local intensity information simultaneously, details of CAC method is explained by Sheppard et al.^50^. The 3D images were segmented to four phases: (1) solid including organic and inorganic minerals, (2) empty fractures (including fracture spaces filled with air), (3) fluid-saturated fractures (filled with either water or oil), and (4) fluid saturated matrix (water or oil that moved from the fractures into the matrix (Fig. 2c).

### Small angle X-ray scattering (SAXS)

SAXS analysis was performed to ascertain the sub-nano level pores in the ORS. A tightly collimated beam of X-rays with known intensity and of fixed wavelength (λ) is scattered on contact with a highly transmitting sample^57^. Such scattering occurs due to the scattering contrast between different constituents residing in the sample. The intensity of the scattered radiation is then measured against the scattering angle (Θ), which is then converted to scattering vector (Q = 4π/λsin(Θ/2)) with units of Å^−1^^58,59^. More in-depth details of SAXS technique can be found elsewhere^57^.

**Figure 3:**
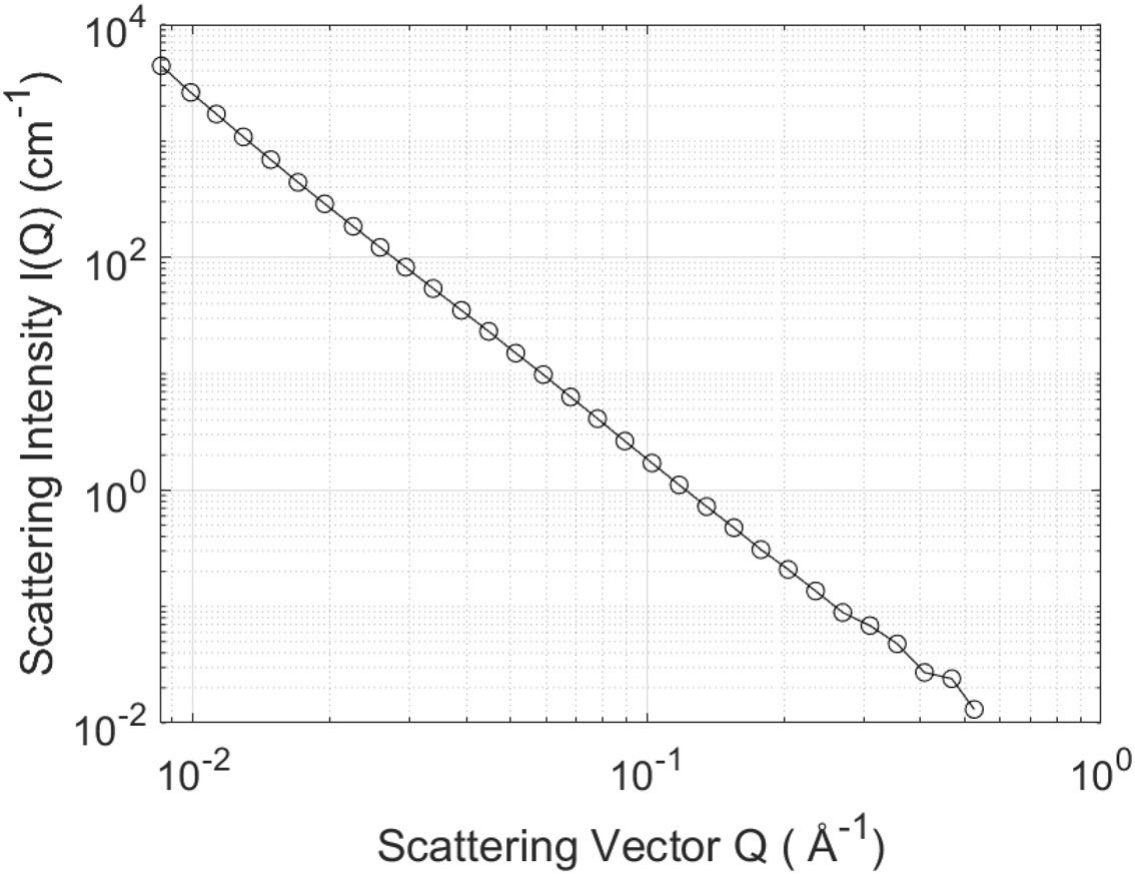
SAXS data for ORS.

SAXS data for each sample was collected for 25 min on an Anton Paar SAXSPoint 2.0 system using a copper (Cu) microfocus X-ray source and Dectris Eiger R 1 M detector. The data was circularly averaged, adjusted for transmission and sample thickness, placed on an absolute scale using water as a secondary standard (where I(0) = 0.01633 cm^−1^ at 25 °C), and had the background subtracted. The data was analysed using the PRINSAS software^60^. The scattering length density (SLD) was calculated to be 2.25 × 10^11^ cm^−2^ using the measured mineral composition from XRD and literature densities. For SLD calculations, the code developed by Paul Kienzle at the National Institute of Standard and Technology (NIST) was utilized (cf. https://www.ncnr.nist.gov/resources/activation/). The details of calculations including the equations used can be found in Hinde^60^. The SAXS data for ORS is shown in Fig. 3.

### Focused ion beam–scanning electron microscopy (FIB–SEM)

The FIB–SEM images of the MVF Beetaloo shale were obtained using the Carl Zeiss AURIGA CrossBeam equipment. A low energy argon ion beam (Hitachi IM4000 ion milling machine) was used to mill a 0.5 mm^2^ surface area of the sample. The sample was platinum coated before being mounted on the equipment. Different spots on the sample were then imaged to check for sub-nano pores and their structure.

## Results and discussion

### Contact angle (CA) and spontaneous imbibition (SI)

**Figure 4:**
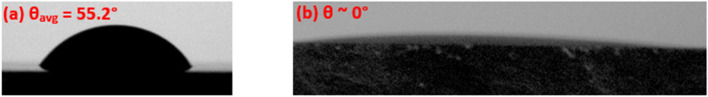
Illustrative examples of (**a**) water droplet CA and (**b**) complete spreading of oil droplet.

As exemplified in Fig. 4, in CA tests, while oil completely spread (~ 0° CA) on the ORS surface (in presence of air), water exhibited a larger contact angle. The average water CA was 55.2° with a standard deviation of ~ 4.1°.

**Figure 5:**
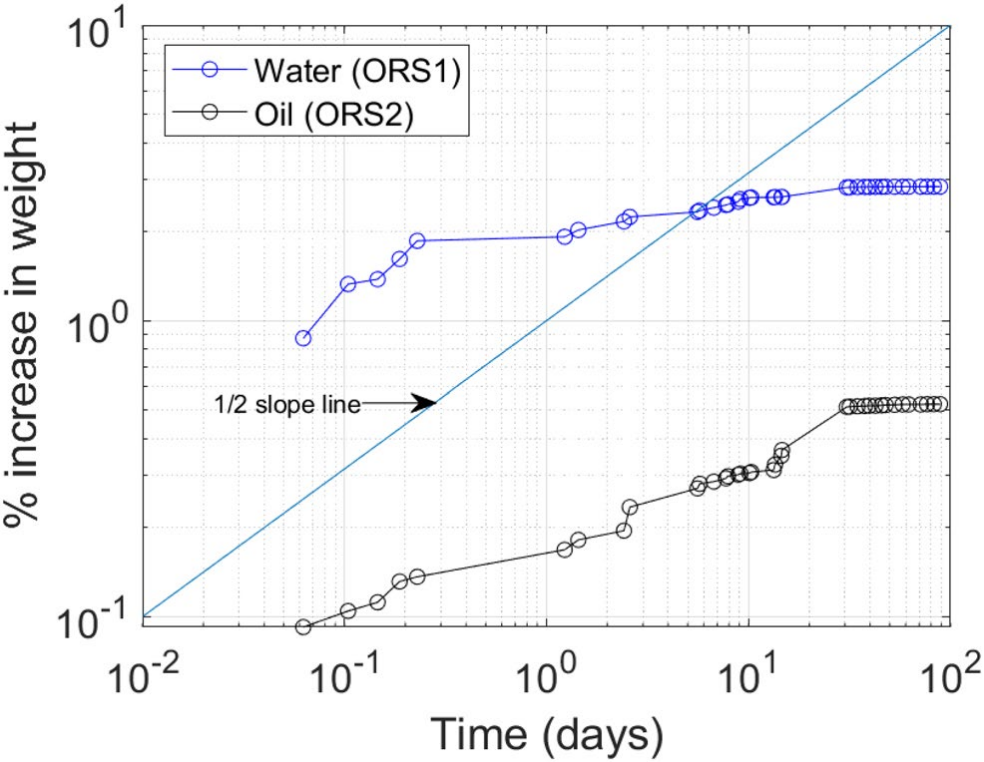
Water and oil uptake (normalized to initial sample weight) in ORS1 and ORS2.

In the conventional definition of capillarity, this implies that during SI tests of each phase in the presence of air, water should imbibe much less than oil. However, in SI tests, the contrary was observed where oil imbibed less than water (Fig. 5). This anomalous observation has been widely reported^6^, yet there is no clarity on why it so happens. It is noted that pure capillary behaviour will scale linearly with t^0.5^ based on the Washburn theory^23^. A half (½) slope line is drawn on Fig. 5 to infer the proportionality of water/oil uptake with t^0.5^. As seen in Fig. 5, only the first few hours of uptake is characterized by dominant capillary process and deviation from ½ slope line begins only after a few hours of the start of fluid uptake. The varying deviations of water and oil signify that different mechanisms are in play in their respective uptakes. Deviations from ½ slope as such occur when boundary conditions begin to influence the fluid movement^61^. Hence, here, such deviation implies the void space in the first continuum (pre-existing fractures) is filled and further uptake is governed by the properties of the second continuum (the matrix including fractures with nano-scale aperture). Water is influenced by further entropic effects (such as osmosis due to clay minerals) and deviation from t^0.5^ is noticeable.

Nevertheless, as discussed earlier, gravimetric curves are unable to provide insights into specific flow dynamics of fluids within the bulk’s porous media. Hence, to “see” what transpires inside the porous media, X-ray and neutron imaging as well as SAXS was performed, analysis of which is discussed subsequently.

### Flows in the first and second continuum

**Figure 6:**
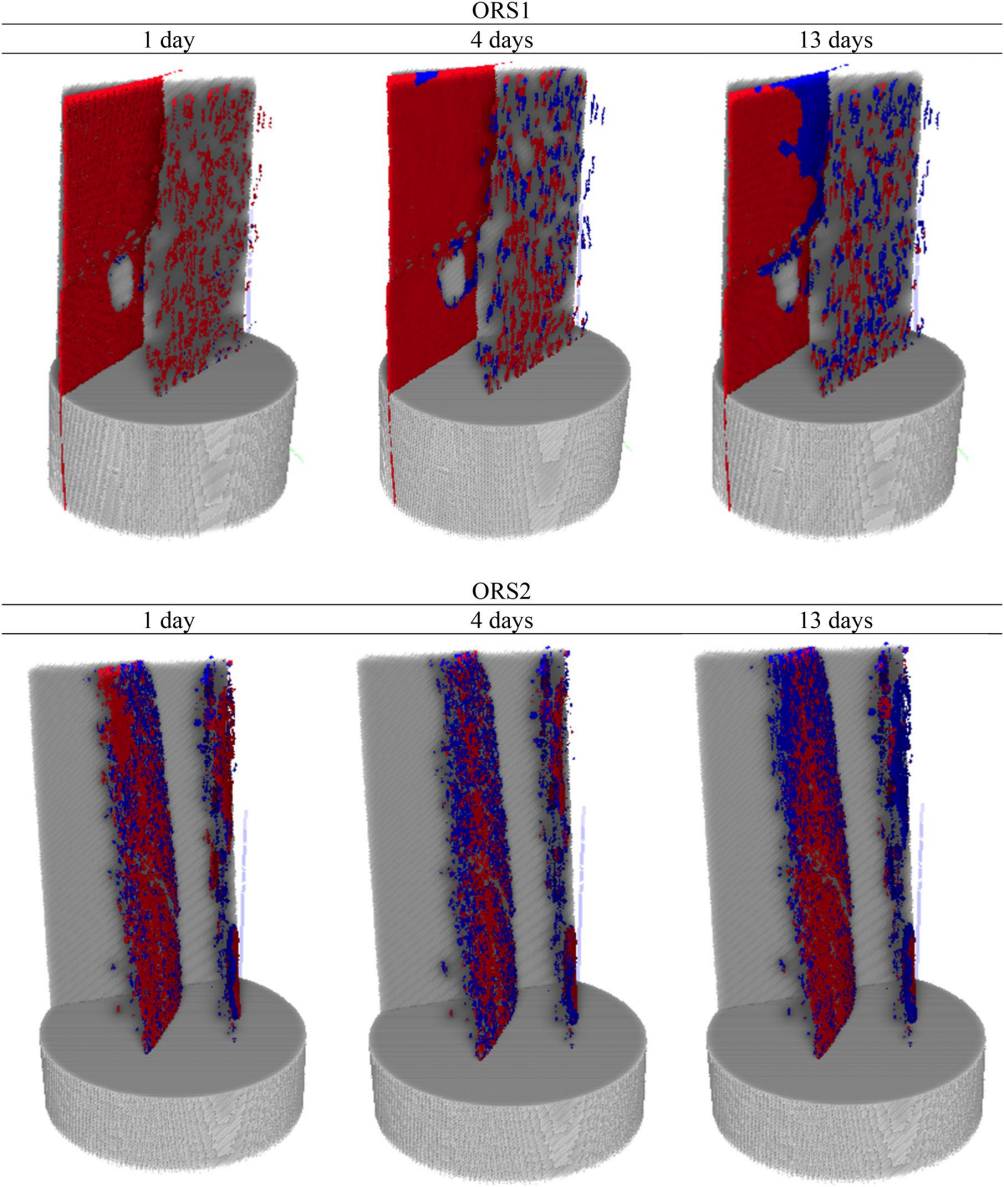
The segmented internal structure of ORS1 (top) exposed to water and ORS2 (bottom) exposed to oil showing empty fracture space (red) and the fraction of fracture space filled with water/oil (blue) at 1 day, 4 days, and 13 days. The red colour represents empty fracture space, and the blue colour represents fluids residing in the fractures.

To assess the oil and water imbibition into the fractures of the shale samples (the first continuum), the 3D neutron images were registered to the 3D X-ray images for both samples ORS1 and ORS2 exposed to water and oil respectively. As discussed, the neutron imaging determines only the hydrogen (water or oil) distribution while the fracture spaces are extracted from X-ray images. It is emphasized that water and oil identified in both continuums are coalescence of molecules as the resolution cannot track single molecular flow^47^. The fraction of the pre-existing fractures filled by oil and water were quantitatively identified from registered and segmented 3D X-ray and neutron images of ORS1 and ORS2 (Fig. 6). As seen in Fig. 6, oil was imbibed in these pre-existing fractures more than water. This is consistent with the results obtained from contact angle measurement showing strong oil wetness (complete spreading) of the sample in the presence of air.

**Figure 7:**
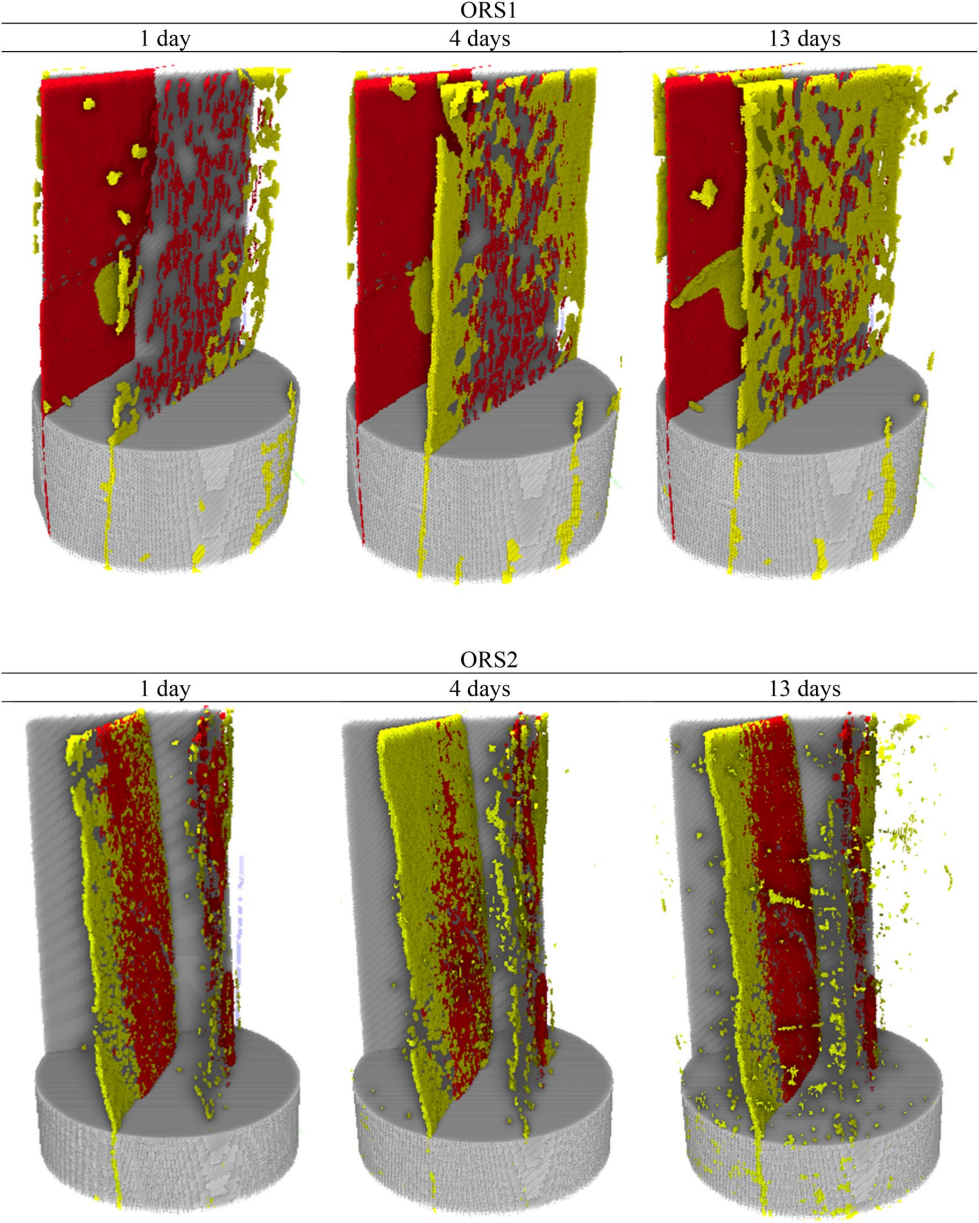
Segmented internal structure of ORS1 (top) and ORS2 (bottom) showing empty plus water/oil filled fracture space (red) and water/oil diffusion into matrix (yellow) at 1 day, 4 days, and 13 days. The red colour represents fracture space plus the fluids residing therein, and the yellow colour represents the fluids diffused into the matrix adjacent to the fractures.

However, the gravimetric curves (Fig. 5) showed that cumulative water uptake was always higher than oil despite having relatively minor hydration induced fractures in ORS1. Hence, it is intrigued if the water is not in the fractures, then where is it? This inquisitiveness is satiated by realizing that there is significant water diffusion into the shale matrix (second continuum) through the fracture planes (Fig. 7 top). Neutron imaging at 1 day showed significant water already diffused into the matrix from the fracture. This is asserted by the water gravimetric curve (Fig. 5) where deviation from ½ slope was already evident before 1 day of water exposure. The similar deviation was also observed for oil, and as seen diffusion of oil also occurred from the fracture to the matrix although at much lower rate (Fig. 7 bottom).

Further, from the segmented 3D X-ray and neutron images, a quantitative analysis was performed. As shown in Table 1, fracture porosity (ratio of empty fracture volume to sample bulk volume) as estimated from X-ray images are 1.925% for ORS1 and 0.365% for ORS2. The fraction of fractures filled with water (ORS1) is 4.07%, 8.74%, and 27.1% after 1 day, 4 days, and 13 days of exposure respectively. On the other hand, the fraction of fractures filled with oil (ORS2) is 30.4%, 44.1%, and 49.2% after 1 day, 4 days, and 13 days of exposure respectively. It is evident that oil was imbibed much higher by the fractures in ORS2 than was water in ORS1. It is noted that relative measurements are of interest to compare between oil and water and actual quantitative value is not of interest i.e. measured within obtained resolution.

**Table 1:** Quantitative analysis from 3D segmented images.

	ORS1			ORS2		
	1 day	4 days	13 days	1 day	4 days	13 days
Fracture porosity	1.925	1.925	1.925	0.365	0.365	0.365
Fluid saturation in fracture	4.07	8.74	27.17	30.39	44.16	49.19
Fluid saturation in matrix (diffused from fracture)	1.21	1.68	3.73	0.29	0.57	0.88

However, when diffusion (flow into second continuum) is quantitatively analysed, it was seen that the void fraction of invaded matrix filled with fluid was 1.2%, 1.7%, and 3.7% after 1 day, 4 days, and 13 days of exposure respectively (Table 1). Contrastingly, oil diffusion into the matrix was just 0.29%, 0.57%, and 0.88% after 1 day, 4 days, and 13 days of exposure. This is also evident from 3D neutron images where significant water diffusion occurred into the matrix (yellow regions) from the fracture (red regions) compared to negligible diffusion of oil (Fig. 7).

### Pore structure and configurational diffusion

As discussed earlier, the fracture system showed oil wetness consistent with CA measurements, however, water diffused to the shale matrix more than oil as seen from Fig. 7. This is contrary to the CA measurement where the shale samples have lower hydrophilicity (in air) (55.2° CA for water versus 0° for oil) i.e. the overall system wettability was strongly oil-wet and slightly water-wet in air. It is known that two sets of distinct pore systems exist in the shale matrix: organic and inorganic. The organic pore system is generally oil-wet^62^. It has been shown that this organic matter must exceed 5% of the total sample weight in order to have strong oil-wet pore connectivity^62^. TOC of 3.15 wt% in the ORS used in this study hence implies that oil-wet pathways in the ORS pore network are likely to be poorly connected for oil uptake. The contribution of oil-wet organic matter porosity to the overall fluid uptake in ORS is thus lower than that of inorganic constitutes which have comparatively connected pathways. The inorganic pore system (which includes clays) can have varying wettability and it remains unknown to what extent it dictates the overall observed wettability behaviour of organic-rich shales^6^. These factors, combined, affect the water/oil uptake by shale. To put things in perspective, there are several mechanisms in play:

1. potential oil uptake, due to capillarity, into oil-wet organic matter that has a low volumetric contribution to the matrix (in this case ~ 3.15 wt%) and limited connectivity

2. potential oil uptake, due to capillarity, into inorganic constitutes that can be oil-wet and have strong connectivity

3. potential water uptake into inorganic pores due to combined capillarity and osmotic forces.

Additional osmotic forces have proven to be responsible for water uptake into inorganic clay constitutes of the shale matrix^5,63^. The electrostatic charges of clay surfaces forming the pore wall (or between the clay interlayers) play a significant role in aqueous fluid flow transport. This phenomenon is directly linked to pore sizes i.e. pore size dictates the effective flow path in the nano-pores. These essentially charged nanopores lead to diffusio-osmosis (a combination of electro- and chemo-osmotic flow of water) that enhances the aqueous solution diffusion process. These phenomena have been well studied in chemical science^64^ where water movement in charged nano-channels has been investigated. Subsurface flow-related studies in this regard also showed that water uptake into shale is influenced by chemical gradient along with capillary forces^65^.

The oil uptake into organic pores due to capillary forces cannot lead to significant oil uptake as the amount and connectivity of organic matters are often limited^62^. It is however not yet clear why the oil is not imbibed into oil-wet inorganic matter, but water is. This cannot be simply due to osmotic forces being stronger than capillarity as many shale samples rich in non-swelling clays, that are expected to develop minimal osmosis, still imbibe more water than oil^5^. Further, it is known that reverse osmotic flow of water out of the shale occurs when the ionic concentration of shale is lower than exposed fluid. However, water uptake into shale (with non-swelling clays) at very high ionic concentration was still significantly higher than oil^5^.

Interestingly, it was observed in Barnett shale using small angle and ultra-small angle neutron scattering (SANS and USANS) that there was restricted methane diffusion in pores with radii < 30 nm whereas water diffused unrestricted into smallest of pores. In larger pores, there was no observed competition for pore accessibility. Also, in another study on Barnett and other shales, it was shown that smaller pores (< 1 nm) were resolved using carbon dioxide (CO_2_) than with nitrogen (N_2_) implying that the pore accessibility of different molecules is unique^66^. Other such studies have also shown that shales can have significant nano-porosity^67^. Therefore, configurational diffusion is shown to construe the anomalous water/oil infiltration process into the shale matrix unexplainable by capillary or osmotic concepts.

**Figure 8:**
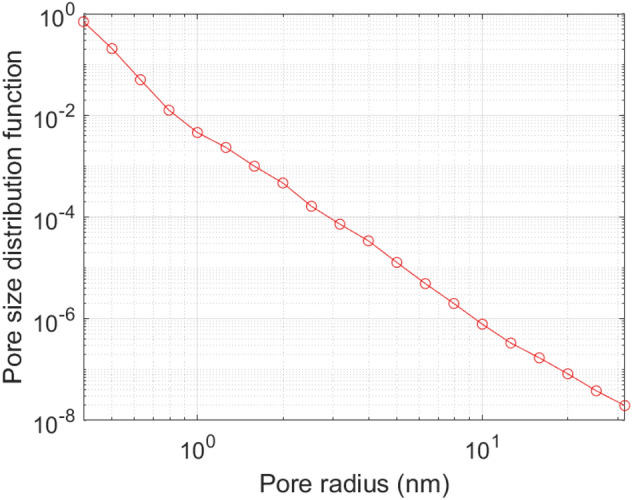
Sub-micron pore size distribution from SAXS for ORS.

**Figure 9:**
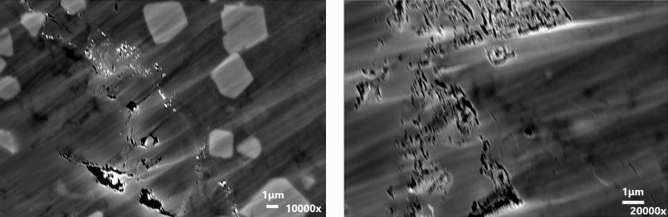
FIB–SEM images of shale sample showing the sub-micron pores.

In order to investigate this further, the pore size distribution of the ORS from 0.3 to 32 nm was measured using SAXS (Fig. 8). The presence of sub-nanometre pores will support the configurational diffusion concept as a cause of restricted flow and storage of oil molecules in them. The results show a significant fraction of pores in the sub nanometre region. These pores are accessible to water (which is about 2.7 Å in size) but become inaccessible to oil molecules with molecular lengths on the nanometre scale for the C_12_–C_14_ iso-paraffins used here. The presence of such pore sizes corroborates the configurational diffusion hypothesis. The lower oil uptake is hence simply due to a physical barrier for effective diffusion of its molecules into the shale matrix compared to water molecules. Configurational diffusion has been well observed and documented in flow of hydrocarbons through zeolite catalysts in chemical science^17,68^. Hence, configurational diffusion of molecules can be significant in such pores. The FIB–SEM images (Fig. 9) also show significant nano-pores present in the shale sample and supporting such behaviour.

Therefore, such observations support our hypotheses that while the mineral and organic constitutes might be strongly oil-wet (in air), they do not have the right pore size to accumulate the oil molecules and allow its physical passage. Water on the other hand, can readily move through nano-scale pores and diffuse faster into the matrix than oil. This will be more pronounced when moving from relatively larger mesopores to micropores^69^. It is noted that rock heterogeneity can affect fluid diffusivity in shale matrix^70,71^, but this was not the focus of the study here. We further employ the non-equilibrium thermodynamics to derive the constitutive equations to investigate whether the theory can also support the observed behaviour.

## Theoretical exposition

A porous medium infiltrated with a fluid can be theoretically characterized in two ways: as a heterogenous medium with distinct mixtures of solid and fluid, or as a single continuum with effective solid and fluid properties^72^. The single continuum viewpoint was previously adopted in Biot’s theory of poro-elasticity^73^. It was further proved efficient in developing many advanced constitutive equations^74^^,^^75^. Hence, the single continuum concept is adopted here. For this, principles of non-equilibrium thermodynamics were employed. The internal energy of a macroscopic region in the porous medium, at isothermal conditions, will change only with the ingress and egress of fluid mass and with the dissipation occurring therein due to fluid movement in respect to solid skeleton. To arrive at fluid transport equations, it is assumed that such dissipation is only due to friction between solid/fluid phases during flow (no chemical reaction occurs) where the macroscopic expression for entropy production is given by:

2 $$0 \le T\gamma = - {\varvec{I}}^{{\varvec{j}}} \cdot \nabla \mu^{j}$$

where $\gamma$ is entropy production rate, $T$ is temperature, ${\varvec{I}}^{{\varvec{j}}}$ is mass flux of *j*th fluid component, where $j = w,o,c$ for water, oil, and any dissolved component (e.g. in water) respectively. The mass flux is given by:

3 $${\varvec{I}}^{{\varvec{j}}} = \rho^{j} \left( {{\varvec{v}}^{j} - {\varvec{v}}^{s} } \right)$$

where $\rho^{j}$ is the component density, ${\varvec{v}}^{{\varvec{j}}}$ its velocity, and ${\varvec{v}}^{{\varvec{s}}}$ is the velocity of the solid phase. It is more convenient to replace the mass fluxes (which are relative to solid velocity) with diffusion fluxes $\left( {{\varvec{J}}^{{\varvec{j}}} } \right)$ that are relative to the fluid’s barycentric velocity $\left( {{\varvec{v}}^{{\varvec{g}}} } \right)$:

4 $${\varvec{J}}^{{\varvec{j}}} = \rho^{j} \left( {{\varvec{v}}^{{\varvec{j}}} - {\varvec{v}}^{{\varvec{g}}} } \right)$$

where ${\varvec{v}}^{{\varvec{g}}} = \sum\nolimits_{j} {\left( {\rho^{j} /\rho^{t} } \right){\varvec{v}}^{j} }$ and $\rho^{t} = \sum\nolimits_{j} {\rho^{j} }$. It is thus obvious that the two types of fluxes are related through:

5 $${\varvec{J}}^{{\varvec{j}}} = {\varvec{I}}^{{\varvec{j}}} - \rho^{j} \left( {{\varvec{v}}^{{\varvec{g}}} - {\varvec{v}}^{{\varvec{s}}} } \right)$$

Here, Darcy velocity is introduced using:

6 $${\varvec{u}} = \phi \left( {{\varvec{v}}^{{\varvec{g}}} - {\varvec{v}}^{{\varvec{s}}} } \right)$$

where $\phi$ is the porosity of the medium. Also, recalling the Gibbs–Duhem equation for a fluid^72^:

7 $$\mathop \sum \limits_{j = w,o,c} \overline{\rho }^{j} \nabla \mu^{j} = \nabla p_{pore}$$

where $\overline{\rho }^{j} = \rho^{j} /\phi$ and the summation can be dropped in case of one fluid system (with no dissolved species). Using Eqs. (6) and (7), Eq. (2) can be further simplified to:

8 $$0 \le T\gamma = - {\varvec{u}} \cdot \nabla p_{pore} - \mathop \sum \limits_{j = w,o,c} {\varvec{J}}^{{\varvec{j}}} \cdot \nabla \mu^{j}$$

For the specific case, when no dissolved components are present in the fluids (e.g. DIW and Soltrol-130), Eq. (8) becomes:

9 $$0 \le T\gamma = - {\varvec{u}} \cdot \nabla p_{pore} - {\varvec{J}}^{j} \cdot \nabla \mu^{j}$$

where now $j = w, o$ for water (DIW) or oil (Soltrol-130) respectively and $\nabla p_{pore}$ is the fluid pressure in the porous medium. In the case of spontaneous imbibition (SI), it is simply the capillary pressure $\left( {\nabla P_{c} } \right)$. From the dissipation, two forces are identified: capillary pressure $\left( { - \nabla P_{c} } \right)$ and chemical potential $\left( { - \nabla \mu^{j} } \right)$. Using the framework of irreversible processes, a linear relation between fluxes and forces exists such that:

10 $${\varvec{u}} = L_{11} \nabla P_{c} + L_{12} \nabla \mu^{j}$$

11 $${\varvec{J}}^{{\varvec{j}}} = L_{21} \nabla P_{c} + L_{22} \nabla \mu^{j}$$

where $L_{mn}$ are phenomenological coefficients where $m,n = 1,2$. Since it was already stated that water (DIW) and oil (Soltrol-130) do not contain any dissolved species, the above equations represent the fluid flux each due to capillarity and diffusion, respectively. The above phenomenological equations are further simplified by assuming that, in the case for fluids with no dissolved species, capillary and diffusive flow due to change in chemical potential is negligible due to constant chemical potential of the fluid i.e. $\nabla \mu^{j} = 0$. It is noted that Onsager symmetry is not invoked here^76^. Onsager’s microscopic time reversibility has been disputed in specific cases^77^ although it may be valid for particular situations^78^. In this case, when the same capillary force can cause different fluxes (either capillary or diffusive) depending on the pore size, it is clear that Onsager’s transport matrix symmetry does not hold.

Next, the phenomenological coefficients $\left( {L_{11} ,L_{21} } \right)$ can be estimated by first realising that capillary flow, such as during SI, can be described as laminar due to its quasi-static nature and hence can be characterized by Poiseuille’s law:

12 $${\varvec{u}}^{{\varvec{j}}} = \frac{{A_{p} r^{2} }}{{8\mu_{j} }}.\nabla P_{c}$$

where $A_{p}$ is the total cross sectional pore area assuming parallel cylindrical pores, $r$ is the pore radius, $\mu_{j}$ is the fluid viscosity, $\nabla P_{c}$ is the capillary pressure gradient across the porous body. Second, under the same capillary pressure gradient, when the fluid molecules encounter pores with openings comparable to their sizes, the flow is then transformed into a diffusion process. The diffusion flow rate of fluid molecules is given by^79,80^:

13 $${\varvec{J}}^{{\varvec{j}}} = \frac{{D_{j} \overline{V}_{j} A_{p} }}{RT}.\nabla P_{c}$$

where $D_{j}$ is the diffusivity of the fluid, $\overline{V}_{j}$ its molar volume, $R$ is the universal gas constant, and $T$ is temperature. The phenomenological coefficients $\left( {L_{11} ,L_{21} } \right)$ are then the coefficients of $\nabla P_{c}$ in Eqs. (12) and (13) respectively. The contribution of capillary and diffusive flow of water towards the total flow can be estimated by taking the relative ratio. Since fluid properties for water are known easily than for oil, we have for water ($j = w$):

14 $$\frac{{{\varvec{u}}^{{\varvec{w}}} }}{{{\varvec{u}}^{{\varvec{w}}} + {\varvec{J}}^{{\varvec{w}}} }} = \frac{1}{{1 + \frac{{8\eta_{w} D_{{{\text{H}}_{2} {\text{O}}}} \overline{V}_{{{\text{H}}_{2} {\text{O}}}} }}{{r^{2} RT}}}}$$

**Figure 10:**
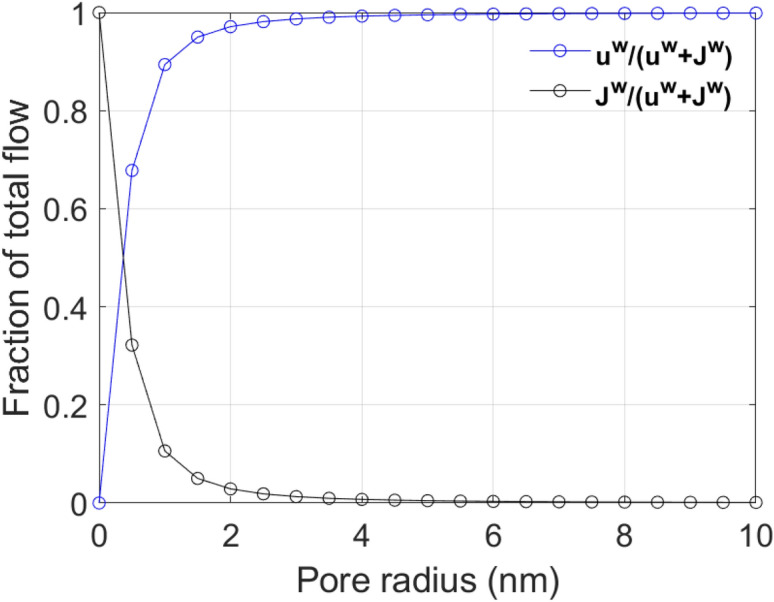
Fraction of total flow due to capillarity and diffusion.

Using the respective average values of diffusivity, viscosity, molar volume, and $RT$ at 25 °C: $D_{{{\text{H}}_{2} {\text{O}}}} = 2.295 \times 10^{ - 9} \,{\text{m}}^{2} {\text{/s}}$, $\eta_{w} = 8.9 \times 10^{ - 4} \,{\text{Pa}}\,{\text{s}}$, $\overline{V}_{{{\text{H}}_{2} {\text{O}}}} = 0.000018\,{\text{m}}^{3} {\text{/mol}}$, and $RT = 2477.6{\text{ J/mol}}$, the ratio of capillary and diffusive flows to the total flow versus pore radius can be generated by solving Eq. (14) (Fig. 10). Figure 10 indicates that the laminar Poiseuille’s flow (or Darcy’s flow) dominates beyond the 2 nm pore size value while below 2 nm, diffusive flow initiates its significance and becomes dominant at small pore sizes (< 0.5 nm).

**Figure 11:**
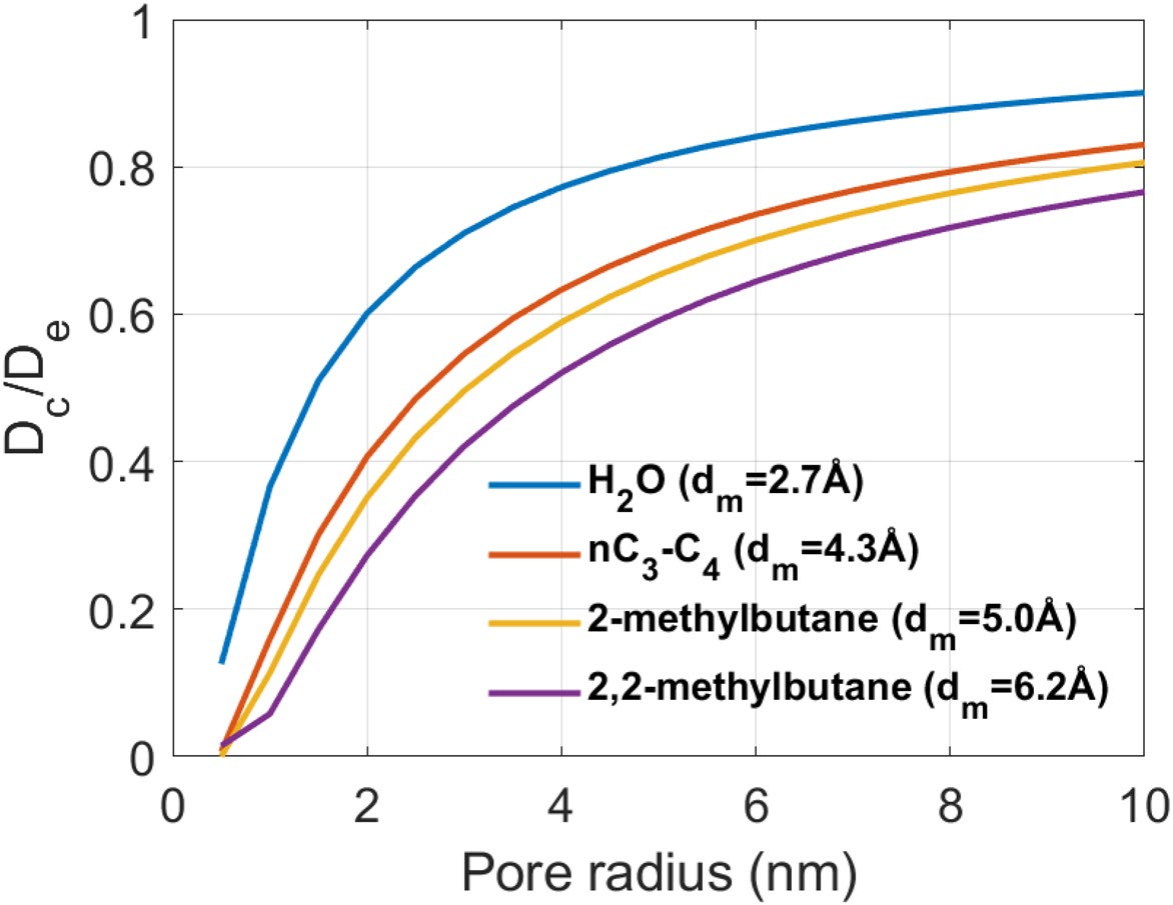
Restricted diffusion versus pore size for molecules of different sizes.

At such small pore sizes where the size of molecules is comparable, configurational diffusion is significant and the easy passage and storage of such molecules in these pores is hindered. When molecular sizes are much larger than the pore size that they are approaching, strong repulsion leads to complete impasse. In fact, in studies related to biological membranes, it was observed that diffusion of molecules is hindered at pore sizes even 20 times larger than the molecular sizes^80,81^. This occurs due to a combination of steric and viscous drag effects. The diffusion coefficient of fluid in a porous medium thus cannot be a single value, rather varying based on the pore size distribution. Below a critical pore size, configurational diffusion is significant and must be incorporated in fluid flow models. A simplified restriction factor for effective diffusion was developed by Pappenheimer et al.^82^:

15 $$\frac{{D_{c} }}{{D_{e} }} = \frac{{\left( {1 - \lambda } \right)^{2} }}{1 + 2.4\lambda }$$

where $D_{c}$ is the configuration diffusion coefficient, $D_{e}$ is unrestricted diffusion coefficient in larger pores and $\lambda = d_{m} /d_{p}$ is the ratio of fluid molecular diameter $\left( {d_{m} } \right)$ to pore diameter $\left( {d_{p} } \right)$. Figure 11 [based on Eq. (15)] clearly shows the reduction of effective diffusion $\left( {D_{c} /D_{e} } \right)$ with pore radius. As seen for different molecules, hydrocarbons always have lower diffusivity than water due to their larger molecular sizes. The molecular sizes of different molecules were taken from Breck^83^. Configurational diffusion is thus more pronounced for heavier and larger molecules such as oil (C_12_–C_14_) than for water. This theoretical exposition corroborates our experimental results and satiates the intriguing question of why oil always imbibes much less than water into shale matrix despite spreading more than water on shale surface.

**Figure 12:**
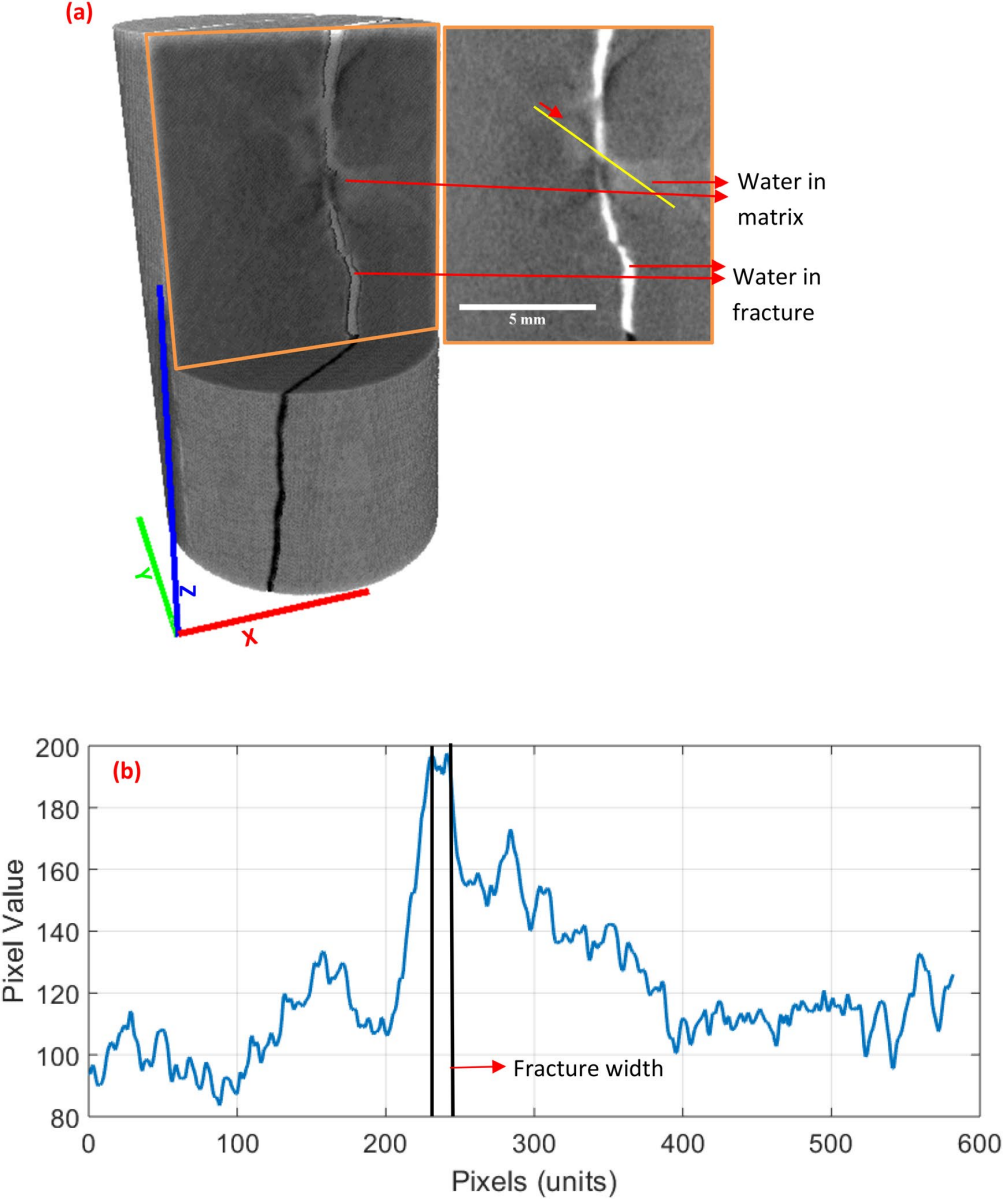
(**a**) Neutron radiograph for ORS1 at 13 days showing water diffusion; the bright white color represents water inside the fracture and the less bright color represents diffused water in the matrix adjacent to the fracture, (**b**) non-homogenous water diffusion on either side of the fracture illustrated by the pixel profile of yellow line drawn across the fracture.

It is noted that the theory of configurational diffusion is based on simplified assumptions such as considering molecules to be spherical and pores cylindrical. It is, therefore, able to predict only part of the impasse of molecules^82,84^. This implies that the configurational diffusion coefficient can be much lower than theoretically predicted values. Also, such a simplified theory is unstable at extremely low pore sizes (< 1 nm). The need for experiments hence is not eliminated. For instance, in realistic shale matrix pore network, larger pores may be preceded by narrower pore throats where configurational diffusion could be significant. Hence, a substantial number of larger pores also remain inaccessible to such molecules drastically lowering their cumulative uptake. This causes heterogenous water diffusion as captured in neutron imaging (shown in Fig. 12 using a 2D neutron radiograph at 13 days (1 pixel = 13.6 µm). As seen in Fig. 12a, the brightest region is inside the fracture where concentration of water molecules is very high. The lesser brighter regions in the matrix correspond to water diffused into the matrix. A yellow line is drawn across the fracture whose pixel profile is shown in Fig. 12b. As can be seen, there is non-uniform diffusion both sides of the fracture. On the left side, ~ 100 pixels are detected with water molecules whereas on the right side, ~ 300 pixels are detected with water molecules. It is reiterated that attributing one diffusion coefficient for such a diffusion mechanism is pointless as there is significant spatial and pore size-dependent variation. Therefore, an accurate theory of configurational diffusion especially in geomaterials is incomplete thus warranting further research. As discussed earlier, new techniques such as PFG NMR^45,46^ and compressed sensing MRI (CS-MRI)^39^ have potential to assist and/or overcome X-ray and neutron imaging limitations and can thus play a role in further strengthening the configurational diffusion theory.

## Conclusion

A combination of X-ray and neutron imaging supported with SAXS and FIB–SEM techniques was used in this study to reveal that higher water uptake by organic rich shale samples against the capillary prediction is due to pore inaccessibility by the larger oil molecules i.e. configurational diffusion of molecules. The theoretical constitutive models were further developed using non-equilibrium concept to support the experimental observations. Significantly smaller water molecules can diffuse through much smaller pore-throats and reside in pores inaccessible by oil. This study highlights that retardation of fluid diffusion due to a subtle phenomenon—the inaccessibility of pores smaller than size of the fluid molecules—that can cause a significant anomaly in theoretically predicted fluid volumes. This further implies improving the theory of fluid uptake in porous media.

It is hence perceived that (1) higher the organic matter content, faster the hydrocarbon diffusion occurs and (2) if oil-wet pores of inorganic matrix constitutes are within nano-scale, the role of capillary is relatively limited especially due to oil molecular size causing low oil uptake whereas water can still diffuse into these nano-scale pore throats assisted by osmotic forces. Therefore, the fewer the micropores exist in the inorganic constitutes of the shale matrix, the higher the rate of oil diffusion will be. These observations are likely to be applicable to fluid uptake in all pores (organic and inorganic) of shale rocks.

## Data Availability

The datasets generated and/or analysed during the current study are available in the Figshare repository (Link: https://doi.org/10.6084/m9.figshare.11860215.v1).
